# Phenotype, Susceptibility, Autoimmunity, and Immunotherapy Between Kawasaki Disease and Coronavirus Disease-19 Associated Multisystem Inflammatory Syndrome in Children

**DOI:** 10.3389/fimmu.2021.632890

**Published:** 2021-02-26

**Authors:** Ming-Ren Chen, Ho-Chang Kuo, Yann-Jinn Lee, Hsin Chi, Sung Chou Li, Hung-Chang Lee, Kuender D. Yang

**Affiliations:** ^1^MacKay Children's Hospital, Taipei, Taiwan; ^2^MacKay Junior College of Medicine, Nursing, and Management, New Taipei City, Taiwan; ^3^Kawasaki Disease Center and Department of Pediatrics, Kaohsiung Chang Gung Memorial Hospital and Chang Gung University College of Medicine, Kaohsiung, Taiwan; ^4^Genomic and Proteomic Center, Kaohsiung Chang Gung Memorial Hospital, Kaohsiung, Taiwan; ^5^Department of Microbiology & Immunology, National Defense Medical Center, Taipei, Taiwan; ^6^Institute of Clinical Medicine, National Yang Ming University, Taipei, Taiwan

**Keywords:** Kawasaki disease, multisystem inflammatory syndrome in children, susceptibility, autoimmunity, immunotherapy, coronavirus disease-19

## Abstract

Coronavirus disease-19 (COVID-19) in children is usually mild but some are susceptible to a Kawasaki disease (KD)-like multisystem inflammatory syndrome in children (MIS-C) in the convalescent stage, posing a need to differentiate the phenotype, susceptibility, autoimmunity, and immunotherapy between KD and MIS-C, particularly in the upcoming mass vaccination of COVID-19. Patients with MIS-C are prone to gastrointestinal symptoms, coagulopathy, and shock in addition to atypical KD syndrome with fever, mucocutaneous lesions, lymphadenopathy, and/or cardiovascular events. MIS-C manifests KD-like symptoms that alert physicians to early recognize and adopt the KD treatment regimen for patients with MIS-C. MIS-C linked to COVID-19 teaches us infection-associated autoimmune vasculitis and vice versa. Studies on genetic susceptibility have identified certain human leukocyte antigen (HLA) locus and toll-like receptor (TLR) associated with KD and/or COVID-19. Certain HLA subtypes, such as HLA-DRB1 and HLA-MICA A4 are associated with KD. HLA-B^*^46:01 is proposed to be the risk allele of severe COVID-19 infection, and blood group O type is a protective factor of COVID-19. The autoimmune vasculitis of KD, KD shock syndrome (KDSS), or MIS-C is mediated by a genetic variant of HLA, FcγR, and/or antibody-dependent enhancement (ADE) resulting in hyperinflammation with T helper 17 (Th17)/Treg imbalance with augmented Th17/Th1 mediators: interleukin-6 (IL-6), IL-10, inducible protein-10 (IP-10), Interferon (IFNγ), and IL-17A, and lower expression of Treg-signaling molecules, FoxP3, and transforming growth factor (TGF-β). There are certain similarities and differences in phenotypes, susceptibility, and pathogenesis of KD, KDSS, and MIS-C, by which a physician can make early protection, prevention, and precision treatment of the diseases. The evolution of immunotherapies for the diseases has shown that intravenous immunoglobulin (IVIG) alone or combined with corticosteroids is the standard treatment for KD, KDSS, and MIS-C. However, a certain portion of patients who revealed a treatment resistance to IVIG or IVIG plus corticosteroids, posing a need to early identify the immunopathogenesis, to protect hosts with genetic susceptibility, and to combat Th17/Treg imbalance by anti-cytokine or pro-Treg for reversal of the hyperinflammation and IVIG resistance. Based on physiological and pathological immunity of the diseases under genetic susceptibility and host milieu conditions, a series of sequential regimens are provided to develop a so-called “Know thyself, enemy (pathogen), and ever-victorious” strategy for the prevention and immunotherapy of KD and/or MIS-C.

## Phenotype of Kawasaki Disease-Like Multisystem Inflammatory Syndrome in Children Different from KD

Coronavirus disease-19 (COVID-19) is usually mild in children (1–3). However, 3–6 weeks after the disease or exposure to persons with COVID-19, some children are affected by multisystem inflammatory syndrome in children (MIS-C) (4–9). Those with MIS-C frequently have gastrointestinal symptoms, coagulopathy, and shock in addition to atypical Kawasaki disease (KD) symptoms with intractable fever, mucocutaneous lesions, lymphadenopathy, and/or cardiovascular events (4–8), which alert physicians to early recognition and adopt the KD treatment regimen for them (4–9). MIS-C occurring 3–6 weeks after contracting COVID-19 suggests that MIS-C is an infection-associated autoimmunity. The life-threatening infection-associated hyperinflammatory syndrome does not completely respond to intravenous immunoglobulin (IVIG) therapy, which is a standard treatment for KD. IVIG plus corticosteroids (4–8) or/and blockade of interleukin-1 (IL-1) or IL-6 action (9) have been used to treat patients with MIS-C. Now the questions remains, who are susceptible, what is (are) the trigger(s), how to predict and differentiate between KD and MIS-C, and which regimen is the optimal therapy for KD or MIS-C based on mechanistic infection immunity?

Kawasaki disease (KD) is a hyperinflammatory febrile vasculitis in children below 5 years of age, with at least four of the five clinical symptoms/signs: skin rashes (>90%), bilateral conjunctival injection (>90%), oral mucosal changes (>90%), peripheral extremity changes, and cervical lymphadenopathy (at least 1.5 cm in diameter), which might develop weeks after a mild respiratory or gastrointestinal symptom (10–12). Those with <4 criteria for KD are classified as incomplete or atypical KD. Children in the extremes of the age spectrum (≤6 months old, or ≥5 years old) tend to have atypical KD associated with delayed diagnosis and treatment (13, 14). Atypical presentation of KD in children may be associated with a higher risk of coronary arteritis because of a delayed diagnosis and treatment (14, 15). KD is previously called mucocutaneous lymph node syndrome by *Tamisaku Kawasaki* in 1974 (10), in regard to vasculitis including coronary arteritis and aneurysm (10–15). The hyperinflammatory response of KD is related to infection, autoimmunity, and/or genetic susceptibility (10–12). KD is more prevalent in East Asia, such as Japan, China, Korea, and Taiwan (10–12, 16–18). The incidence of KD varies from country to country, e.g., 4.5 per 100,000 children younger than 5 years of age in India, 25 per 100,000 in the USA, 56 per 100,000 in Taiwan, and over 250 per 100,000 people in Japan (17, 18).

Recently, a surge in the prevalence of KD-like illness in children has been found with the COVID-19 outbreak in the USA, UK, France, Spain, and Italy (4–9, 19). COVID-19 can cause acute respiratory distress syndrome (ARDS), carditis, thrombosis, and/or shock in adults, but generally, induce mild symptoms in infants and children (1–3). The COVID-19-associated MIS-C occurs in older children and tends to manifest with gastrointestinal symptoms, coagulopathy, and shock in addition to the KD symptoms. Patients with KD usually have thrombocytosis (10–15), but patients with MIS-C have high, normal, or low platelets (4–9), which may be related to coagulopathy or microangiopathy. The MIS-C is similar to KD shock syndrome (KDSS) occurring in relatively older children with atypical KD, showing shock, thrombosis, and IVIG resistance (20–22). The immune response in MIS-C is different from that in COVID-19. COVID-19 is contagious but MIS-C is not. MIS-C is due to post-infection autoimmunity because it occurs 3–6 weeks after the exposure to COVID-19 or persons with COVID-19. Patients with MIS-C have a unique serology with anti-S antibodies (IgG, IgM, and IgA) but not anti-N antibodies, in contrast, patients with COVID-19 have both anti-S and anti-N antibodies (23). The skin, gastrointestinal, and shock symptoms in MIS-C are sometimes undifferentiated from those in toxic shock syndrome (TSS), but the medical history is different because TSS is related to superantigens of bacteria which are usually associated with bacterial infections, surgical wound, or usage of tampons (24).

The COVID-19-related MIS-C, representing atypical KD syndrome in older children at a median age of 8 years (6, 9), is prone to IVIG resistance and life-threatening cardiovascular events, such as myocardial infarction, thrombosis, and/or shock (4–9, 19), which is the most life-threatening morbidity in children during the COVID-19 pandemic.

## Similarities and Differences Among KD, KDSS, and MIS-C

There are some overlapped and different symptoms and signs among KD, KDSS, and MIS-C in regard to age, sex, race, severity, and treatment responses (Table 1). Patients with KD usually have vasculitis in mucocutaneous regions (>90% of eyes, lips, and/or skin symptoms) and coronary arteritis, but few patients have myocarditis (~5%) and shock (~7%) (10–12). In contrast, KDSS frequently shows myocarditis, thrombosis, and shock (20–22). The KDSS is associated with a higher rate of IVIG treatment resistance, with older age and more serious hypotension, skin rash, leukocytosis, neutrophilia, and hypoalbuminemia, especially frequently found in Hispanics (20, 22). Patients with MIS-C more frequently have a shock, myocarditis, thrombosis, and gastrointestinal symptoms (4–9). Both patients with KDSS and MIS-C more frequently require intensive care supports, such as inotropic agents, ventilation support, anti-thrombotic therapy, and additional anti-inflammatory therapies (4–9, 19–22). KD is prevalent in Eastern Asia (10–12) but MIS-C is more frequently found in Western countries, especially Afro-Caribbean (4–9, 19). The mean age of patients with KD is 2.5 years, and that of patients with KDSS is 3.7 years (22). In the largest cohort report of 186 Afro-Caribbean patients with MIS-C, the median age is 8.3 years (9), showing prominent gastrointestinal symptoms and thrombosis with variable platelet counts (high, normal, or low).

**Table 1:** Demographic data and phenotypes of Kawasaki disease (KD) and multisystem inflammatory syndrome in children (MIS-C).

**Phenotypes**	**KD**		**COVID-19 Mild in Children**	**MIS-C Multisystem**
	**KD**	**KDSS**		
Skin/mucosa (%)	>90	>90	Few	50–60
CAL (%)	20	65	Rare	29–80
Myocarditis (%)	5	20	Rare	65
Shock (%)	7	100	Rare	60
Age range (Median) (year)	0.5–5.0 (2.0)	2–12 (3.5)	0–18	2–18 (8.3)
Sex	Male	Female	Both	Both
Race	Asian	Hispanic	All races	Afro-Caribbean
IVIG resistance	15%	40%	–	80% (add on steroid)
Fatality (%)	<0.1	0–6.8	<0.1	0–10 (2)
Pathogen	Unknown	Unknown	SARS-CoV-2	SARS-CoV-2 associated

*KDSS, Kawasaki disease shock syndrome; COVID-19, coronavirus disease-19; CAL, coronary artery aneurysm; IVIG, intravenous immunoglobulin; SARS-CoV-2, severe acute respiratory syndrome coronavirus-2*.

In fact, KDSS is a severe form of KD with hypotension, coagulopathy, more cardiovascular involvement, and IVIG resistance, initially recognized in 2009 (20). In a retrospective analysis of 103 patients with KDSS, abnormalities in the coronary arteries were 65% and the mortality rate was 6.8% (22). Before the institution of IVIG therapy for KD, the KD mortality and cardiac morbidity were 2 and 20%, respectively. After the institution of the IVIG treatment for KD, the mortality decreased below 0.1% and coronary artery aneurysm downed to <4%. The MIS-C mortality ranges between 0 and 10% (average, 2%). KD is not contagious although many infections, such as *Staphylococcus aureus*, streptococci, rhinovirus, coronavirus, enterovirus, chlamydia, or Epstein-Barr (EB) virus had been associated with KD. About 40% of patients with KD have reactive skin erythema and/or scaling at the Bacillus Calmette–Guérin (BCG) inoculation site (11), suggesting the autoreactive antigen for KD may cross-react with the antigen of BCG, or the BCG reactivation is a bystander of hyperinflammatory reaction of KD. It is highly suspicious that the MIS-C is a severe KD-like vasculitis mediated by a COVID-19-induced autoimmune reaction. This is not the first time that human coronavirus (HCoV) is correlated to KD. In 2005, Esper et al. (25) reported that a novel human coronavirus called human coronavirus New Haven (HCoV-NH) was associated with an outbreak of KD in New Haven, showing RT-PCR detection of the positivity at 8/11 vs. 1/22 in a case-control study. This association of KD with HCoV was not replicated in a study at Taiwan using 53 consecutive KD samples in which no detectable HCoV-NH or HCoV-NL63 was observed in nasopharyngeal secretions (26). They, however, did not measure the serum antibodies against HCoV-NH or HCoV-NL63. These studies suggest that children in Western countries are susceptible to coronavirus-related KD-like vasculitis and children in Asian countries are susceptible to non-coronavirus-related KD vasculitis.

In laboratory data as shown in Table 2, patients with KD tend to have thrombocytosis, and patients with KDSS or MIS-C tend to have varied platelet counts. C-reactive protein (CRP) and procalcitonin levels are much higher in patients with KDSS or MIS-C. The ferritin levels are also higher in patients with KDSS or MIS-C. Lymphopenia is often prominent in patients with COVID-19 or MIS-C (1–9, 19), but not in patients with KD or KDSS (10–12, 20–22). Coagulopathy is also more often in KDSS or MIS-C (9, 19–22, 27, 28). Ferritin (>500–1,000 ng/ml) and D-dimer (>1,000–4,000 ng/ml) levels are much higher in children with KDSS and MIS-C (9, 19–22, 27, 28). Cytokine storm in the blood is quite similar between KD and MIS-C, showing augmented hypercytokinemia in IL-6, IL-10, IL-17, inducible protein-10 (IP-10) (CXCL10), and MCP-1 (CCL2), especially higher IL-6 and IL-10 levels in KDSS (27), and IL-10 and TNF-α levels in MIS-C (28). Over 80% of patients with MIS-C have detectable anti-S antibody against spike (S) antigen of severe acute respiratory syndrome coronavirus-2 (SARS-Cov-2), but less than one-third have detectable RNA of the virus (4–9). Apparently, MIS-C is mediated by a skewed immune response toward T helper 17 (Th17) reaction in the convalescent stage of COVID-19.

**Table 2:** Laboratory data of KD, COVID-19, and MIS-C.

**Laboratory data**	**Kawasaki disease**		**COVID-19 mild in children**	**MIS-C multisystem**
	**KD**	**KDSS**		
Platelets	>350,000/μl	High or Low	Normal	High, normal, low
CRP (mg/dl)	50–150	>100	<50	>100
Procalcitonin (ng/ml)	>0.5	>1.0	<0.25	>1.0
Ferritin (ng/ml)	100–200	500	<100	>1,000
Lymphopenia	Rare	Rare	Some	Moderate
Coagulopathy	No	Some	Rare	Often
D-dimer (ng/ml)	<1,000	>1,000	<1,000	>4,000
Cytokines	IL-6, IL-17, IP-10	IL-6, INFγ, IL-10	IL-6, IFNγ, IL-8	IL-6, IP-10, IL-10
Anti-S antibody (%)	NT	NT	100	>80

*KDSS, Kawasaki disease shock syndrome; IL, interleukin; IFN, Interferon; IP, inducible protein; NT, not tested*.

## Immunopathogenesis of KD and MIS-C

A patient with a viral infection usually has normal or lower leukocyte counts, and low CRP and procalcitonin levels unless he/she also has superimposed bacterial infections. However, regardless of whether COVID-19 is contagious or MIS-C is not contagious, lymphopenia and elevated CRP are found in both conditions in children. Apparently, SARS-CoV-2 induces a proinflammatory reaction in the acute stage of COVID-19 and a hyperinflammatory reaction of vasculitis (4–9, 19) with augmented levels of Th17 and Th1 mediators in MIS-C (28). There are many unsolved issues on the immunopathogenesis of KD and MIS-C. It is debatable whether MIS-C and KD are post-infectious hyperinflammation, autoinflammatory, or autoimmune disorders (29–34). Current autoimmune concepts have limitations to explain the pathogenesis of variant systemic vasculitis syndrome, which is not contagious but infection-associated hyperinflammation in the convalescent stage (29). Inflammation-inducing substances, not only those originating from pathogens, including toxins and pathogen-associated molecular patterns (PAMPs), but also those originating from injured or infected-host cells including pathogenic proteins, pathogenic peptides, and damage-associated molecular patterns (DAMPs), especially in intracellular pathogen infections, such as virus, chlamydia, BCG, and SARS-CoV-2, may alter the immune responses based on “the protein-homeostasis-system hypothesis” (30). Given the fact that marked different incidences in KD and MIS-C across the populations may be explained by colonization states of pathogens (31), and an imbalance of regulatory and cytotoxic SARS-CoV-2-reactive CD4 T cells in COVID-19 (32), we focused on the imbalanced Th17/Treg regulation for explanations of the same and different manifestations among KD, KDSS, COVID-19, and MIS-C in this perspective article.

We have studied the immune responses of KD for over two decades and look into those related to COVID-19 in the literature in order to explore the link and implication for effective prediction, prevention, and treatment of KD and MIS-C. Literature and clinical experience of KD management prompt early recognition of the KD-like vasculitis in MIS-C for IVIG immunotherapy and beyond, vice versa, information accumulated in MIS-C suggest that KD-like vasculitis is an infection-associated autoimmunity (32–34). Another possibility is an antibody-dependent enhancement (ADE) of FcγR-mediated autoimmunity that has been reported in SARS-CoV-2 infection (35) and also reported in SARS-CoV-1 infection (36). In a genetic association study of FcγRIIA polymorphisms with the severity of SARS-CoV-1 infections, Yuan et al. (37) found that variant FcγRIIA-R/R131 in the intensive care unit (ICU) subgroup of patients with SARS was significantly more frequent than in normal controls. We have previously studied immunopathogenesis of SARS-CoV-1 infection and found that SARS-CoV-1 infection caused an early innate augmentation with adaptive immunosuppression and then induced a late exacerbation (38, 39). To address the hyperinflammatory reaction of KD, we first demonstrate that overexpression of inducible nitric oxide (NO) synthase associated with elevated blood NO levels is present in patients with KD before IVIG therapy (40), and that the T-cell activation marker CD40L is prominently expressed on T cells and platelets in children with KD, which is reversed after IVIG therapy (41). This is comparative to the dynamic time course of immune responses of KD validated by a kinetic immunopathology in a series of autopsy classifications of early necrotizing vasculitis with innate phagocyte activation followed by a remodeling of adaptive immunity with lymphocyte infiltration in the convalescent stage (42).

Before the era of IVIG therapy, the rate of coronary artery aneurysm was 25% and mortality was 1–2% (10, 42–44) in patients with KD, after the institution of IVIG therapy the rate of coronary artery aneurysm downs to 3–4% and mortality to <0.1% (42, 43). Now the challenge for the KD treatment is IVIG resistance in 15–20% of KD patients, which requires additional immunotherapy. Similarly, KDSS and MIS-C have even higher IVIG resistance rates and more frequently require combined therapy with IVIG and steroid pulse therapy (19–22, 28). Interestingly, corticosteroids treatment before the IVIG institution era showed an exacerbated morbidity of coronary artery aneurysms in 64.7 vs. 20% treated with antibiotic alone or 11% treated with aspirin alone (44). Parameters in patients with IVIG resistance are persistent fever and elevated IL-6 levels (45). Furthermore, skewed T-cell polarization toward Th2 response favors the outcomes of IVIG therapy. A higher eosinophil count associated with a higher IL-5 level is a favorable marker for the success of IVIG treatment. In contrast, lower initial eosinophil counts and lower IL-4 and IL-5 levels are associated with IVIG-resistance (46). Patients with KD have prominent Th17 immune responses with augmented IL-6, IL-10, G-CSF, and IL-17A levels, and lower Treg pathway transcription factor FoxP3 expression before IVIG treatment (47). The augmented cytokine storm declines and the Treg cell increases after IVIG treatment.

The Th17 polarization with elevated IL-6, IL-17A, and G-CSF levels is correlated to a higher neutrophils vs. lymphocytes (N/L) ratio in KD patients complicated with IVIG resistance and coronary arteritis (48), which is similar to the severity of COVID-19 associated with an increase in neutrophils and decrease in lymphocytes and elevated INF-γ, IL-6, and IL-8 levels (49, 50). The cytokine profile in MIS-C is different from that in severe COVID-19 in higher IL-10 and TNF-α levels (28). It is shown that IL-6 together with TGF-β induces Th17 differentiation from naïve T cells (51), whereas IL-6 inhibits TGF-β-induced Treg differentiation *via* degradation of FoxP3 (52), suggesting that higher IL-6, IL-10, and IL-17A but lower FoxP3 and TGF-β expression in patients with KD or MIS-C is involved in the Th17/Treg imbalance. This is further supported by our finding that DNA polymorphisms of TGF-β-signaling pathway genes, e.g., TGF-β2 and SMAD3, were associated with the susceptibility of KD (53), and the Th17/Treg imbalance could also be mediated by epigenetic regulation of DNA methylation and/or micro RNAs (miRNAs) on innate and adaptive immune genes as a biomarker of KD (54–59). In HumanMethylation450 BeadChips assay, we have found that DNA hypomethylation on the promoter CG sites of many immune activation genes in leukocytes of patients with KD before IVIG treatment (54–57). The hypomethylated genes were associated with augmented gene (mRNA) expression, particularly the toll-like receptors (TLRs). The TLR1, 2, 4, 5, 8, and 9 receptor genes were significantly hypomethylated and associated with augmented mRNA expression (55). Similarly, other innate immunity genes, e.g., FcγR2A, IL-10, and S100A8 were also hypomethylated before IVIG treatment (54–57). Importantly, we found that the CpG site methylation changes >20% in the acute stage of KD were mainly hypomethylated (97%) genes but only 3% hypermethylated genes (56). After IVIG treatment, the hypomethylated genes and augmented mRNA expression reversed (54–57).

Moreover, it is found that miRNA expression is also a good biomarker of KD, which differentiated KD from other febrile diseases by a set of 4 miRNA expression at C_T_ (miR-1246)-C_T_ (miR-4436b-5p) and C_T_ (miR-197-3p)-C_T_ (miR-671-5p) (58). The miRNA control of Treg expression in patients with KD has been characterized before and after IVIG treatment (59). The epigenetic control of Treg development and maintenance has been defined predominantly *via* FoxP3 expression (60). These epigenetic profiles and functional markers of different Treg population (tTreg, iTreg, and pTreg) tend to have a promising role as specific mechanistic biomarkers for the prediction and prevention of Th17-mediated autoimmunity (61–63). The study model can be applied to study the epigenetic biomarkers and therapeutic targets of MIS-C and KD with and without shock syndrome by potential immunotherapy of cytokine inhibitor, DNA methylation, and/or miRNA expression in addition to IVIG with and without corticosteroids. The immunopathogenesis of KD and MIS-C probably progresses from an early Th17 response, followed by a later T-regulatory response. In the early Th17 response before IVIG treatment, the Treg pathway signals are depressed, and the reciprocal Th17/Treg imbalance reverses after IVIG treatment (47, 54–59). This is supported by the fact that the corticosteroids treatment alone in the acute stage was useless and even harmful in the 1970s (10, 44); instead, the combination of IVIG with corticosteroids showed a better response than the IVIG therapy alone in the 2010s, especially in the patients with IVIG resistance (64). Based on the rationales described above, we postulate that there are two phases during the development of KD or MIS-C syndrome; the early Th17 reaction and late Treg resolution stage have different immunopathogenic processes with individual biomarkers and require different immunotherapies.

## Both KD and MIS-C Occur in Children and Adults

A population-based surveillance system called COVID-19–associated hospitalization surveillance network (COVID-NET) analyzed 576 hospitalized COVID-19 pediatric patients and showed the prevalence of COVID-19 in children increased from 0.1 per 100,000 to 8 per 100,000 with the progress of COVID-19 pandemic, in which a race disparity in hospitalization deviated to Hispanic children, and nine (10.8%) of 83 admitted children had MIS-C (65), suggesting MIS-C may attribute to one-tenth of the admitted severe COVID-19 in children. MIS-C is rare or sporadic in adults (66, 67). COVID-19 deserves further studies on the autoimmunity under endogenous or exogenous milieu because it might directly trigger autoinflammatory conditions by molecular mimicry or cause autoimmunity in predisposed individuals in other environmental conditions (68). The algorithm for diagnosis and treatment of complete and incomplete KD in children has been proposed to diagnose and treat KD in adults (67, 68), and MIS-C in adults (67, 68).

Some adults have been diagnosed with atypical or incomplete KD, contemporarily or retrospectively (69, 70), and occasionally caused sudden death (71) or sequelae of the KD from Children (72). MIS-C has also been demonstrated in certain adults (67, 68). The autopsy of patients with COVID-19 showed a severe endothelial injury associated with the detectable intracellular virus, disrupted cell membranes, and widespread thrombosis with microangiopathy in the lungs (73, 74). The alveolar microthrombi were nine times more in patients with COVID-19 than in patients with influenza (75), and viral particles were detected in epithelial cells and endothelial cells of the lungs (73, 74). Micro-embolism and thrombosis indicating vasculitis and coagulopathy are similar between fatal patients with COVID-19 with ARDS and fatal patients with SARS (75, 76). Pathological findings of autopsy in KD are vasculitis with leukocyte infiltration, called periarteritis nodosa-like arteritis, coronary thrombosis with macrophage and lymphocyte infiltration, and aneurysm, but not pulmonary vasculitis (42, 77, 78). Autopsy features in MIS-C have not been characterized yet. We anticipate that abnormal proinflammatory insults with skewed Th17/Treg imbalance in MIS-C will be seen in the lesions of the cardiovascular system but not in the lesions of pulmonary vessels. The pathology in the COVID-19-induced ARDS showing pulmonary involvement with detectable viral RNA and thrombo-emboli in autopsy is different from the pathological finding in KD showing sterile vasculitis, leukocyte infiltration, and aneurysm, indicating immunity to infections in the former vs. autoimmunity in the latter, which could occur in both children and adults.

## Genetic Susceptibility of KD and MIS-C

No specific genes have been linked to the susceptibility of MIS-C. The siblings of patients with KD are 10 times more likely to have KD. Several susceptibility genes (e.g., *ITPKC, CASP3, CD40*, and *ORAI1*) have been linked to KD (79), and KD is also associated with the human leukocyte antigen (HLA)-BW22J2 subtype, which is found specifically in Japanese and not in Caucasians (80). In patients with KD with coronary artery lesions (CAL), the frequency of HLA-DRB1^*^11 is significantly increased and that of HLA-DRB1^*^09 is decreased (81); In fact, HLA subtypes linked to KD are different between children in Asia and those in Western countries (82). We have previously found that HLA-DRB1 was associated with KD susceptibility (83). To search for the risk allele(s) of major histocompatibility complex (MHC) class 1, HLA-MICA (MHC class I chain-related gene A) locus, we found that the HLA-MICA A4 allele was significantly associated with the coronary artery aneurysm in patients with KD (84), and it has been validated in a genome-wide association case-control study in a Taiwanese population (85). We also found that DNA polymorphisms of TGF-β2 and SMAD3 are associated with the susceptibility of KD (53), and a dominant T allele of rs2243250 in the IL-4 gene conferred a great protective effect against the development of CAL in patients with KD (*p* = 0.006) (86). Taken together, this suggests that a specific HLA subtype could present a viral antigen peptide to T cells and induce a skewed Th17-Th1/Th2-Treg development involved in the altered hyperinflammation in KD. Although there is no any genetic association with MIS-C in the literature, the HLA-B^*^46:01 has been proposed to be associated with the severity of COVID-19 in a computational simulation by using SARS-CoV-2 whole-genome peptides for simulating their binding to 145 MHC class I HLA-A, -B, and -C genotypes, in which HLA-B^*^15:03 shows the greatest capacity to present highly conserved SARS-CoV-2 peptide which is shared among common human coronaviruses, suggesting that it could enable cross-protective T-cell-based immunity (87). Deletion or mutation of TLR7, which is a single-stranded RNA virus sensor in endosomes for induction of interferons (IFNs), contributes to the severity of COVID-19 in young adults (88). In contrast, imiquimod, a TLR agonist is proposed to enhance the defense against COVID-19 (89). Taken together, we would propose to clarify whether an HLA subtype, such as HLA-B^*^46:01 together with a TLR7 variant induces an augmented proinflammatory reaction under conditional milieu and alters the epigenetic control of FoxP3 expression resulting in the Th17/Treg imbalance in KD and/or MIS-C.

Interestingly, different blood group subtypes have also been shown to be associated with the susceptibility of COVID-19 (90–92). Initially, a report from Wuhan, China described blood group A subjects were more susceptible to COVID-19, and the presence of anti-A antibody was probably protective (90). Furthermore, another report from China showed that females but not males, with blood group A are susceptible to COVID-19 (91). In contrast, later in the USA, the other report described that B and AB blood groups were susceptible to the infection but not severity (92). The reproducible result among the studies is that the O blood group population is less susceptible to COVID-19 (90–92), but it remains controversial whether blood groups A and/or B population are susceptible to and/or vulnerable to severity. Perhaps, the differences are related to different studies in different races.

We are currently studying the association of MHC genotypes, haplotypes, and antigen presenting pocket prediction with KDSS and/or MIS-C *via* a consortium in Taiwan. Hopefully, the more the cases of MIS-C identified the more the opportunity to identify the association of KDSS or MIS-C with HLA subtypes in the COVID-19 outbreak or the COVID-19 mass vaccination. We could also study whether different H2 subtypes interacting with viral antigen under host situations lead to altered immunity contributing to KDSS or MIS-C in a mouse model using vaccine antigens with and without adjuvant. This experimental study would test whether the HCoV associated KD-like hyperinflammation is related to different races with varied HLAs by which different antigens induce altered immune responses because of different HLA subtypes and environments.

## Evolution of Immunotherapies for KD and MIS-C

Since MIS-C reveals KD-like syndrome fulfilling complete or incomplete criteria, a physician could rapidly recognize and adopt the treatment regimen of KD for MIS-C, and mitigate the life-threatening disease. The treatment of KD in the acute febrile stage has evolved from corticosteroids, IVIG, and aspirin to a combination of IVIG, aspirin, and steroids through the past 50 years (10, 44, 64). Although long-term aspirin, whether it is high (anti-inflammatory) or low (antiplatelet) dose, does not appear to lower the frequency of coronary abnormalities (42), a low dose aspirin and/or antithrombotic treatment with low molecular weight heparin or warfarin is prescribed according to the progress of coronary aneurysm in the convalescent stage (42, 93). A combination of IVIG and corticosteroids significantly reduced the risk for coronary artery lesions compared with IVIG alone (7.6 vs. 18.9%; OR: 0.3; 95% CI 0.20–0.46) in a meta-analysis (64). Different dosing of IVIG for KD has been clarified (94), and different dosing of corticosteroids in the clinical trials at different countries explained the overall varied benefits on the outcomes of IVIG and corticosteroids in coronary artery aneurysm (64, 95). In pneumonia-associated ARDS, early treatment with corticosteroids and/or IVIG may reduce the aberrant immune responses that have been described (30). This may be also applicable to the treatment of COVID-19-related ARDS. For instance, a combination of pulse corticosteroids and IVIG therapy has been shown to rescue patients with tocilizumab-resistant severe COVID-19 (96). High dose IVIG regimen (2 gm/Kg) is largely demonstrated as more effective (42, 94), however, early IVIG therapy for KD within 4 days did not provide better protection from the development of CAL (97, 98). Whether the earlier and higher doses of immunotherapy for MIS-C and KD responsible for better outcomes deserves further studies.

Intravenous immunoglobulin resistance in the acute stage is frequently associated with the development of CAL in patients with KD. A few different scoring systems have been developed to predict IVIG resistance (99–101), and to provide a precise anti-inflammatory regimen, such as to infliximab (or anakinra) in addition to aspirin, IVIG, and corticosteroids therapy (101). Unfortunately, a scoring system (Kobayashi Score) which is successfully used to predict and prevent CAL in Japan (99) performs poorly in sensitivity and specificity in Western countries (100). MIS-C is IVIG resistant in most patients, therefore IVIG plus corticosteroids is used to treat the life-threatening condition (4–9, 96).

Certain unique complications of KD, such as shock, macrophage activation syndrome (MAS), or coronary aneurysm are usually associated with IVIG resistance and require additional anti-inflammatory regimens, such as cyclosporin, anti-IL1, or anti-IL6 treatment (101, 102). It is reasonable to add anti-IL6 for KD or MIS-C for IVIG resistance because serum IL6 levels correlate with IVIG resistance (45, 103). However, in a study of four patients with IVIG-resistant KD who are responsive to anti-IL6 treatment but affected by coronary artery aneurysms in 2 of them (104). The patients with KD with IVIG-resistance usually respond to anti-TNF, anti-IL-1, or steroid pulse therapy (101–104). Furthermore, anti-cytokines, such as tocilizumab and anakinra or anti-coagulopathy regimens have been used for COVID-19 hyperinflammatory syndrome in adults and resulted in favorable outcomes (105, 106), and are suggested to be used in MIS-C with IVIG resistance (9, 19, 28). Apparently, a scoring system based on symptoms and biochemistry to predict IVIG resistance may not be enough. Some studies had identified IVIG resistance associated with elevated IL-6, IL-10, and/or TNF-α levels in KD with and without shock syndrome (27, 45, 48), and we showed that lower IL-5 levels associated with lower eosinophil number was correlated to IVIG resistance (46), and allele rs2243250T of the IL-4 gene conferred protection against coronary artery lesions in KD (85). Patients with KD or MIS-C in different countries or races may require varied criteria for the prediction of the resistance to IVIG or anti-cytokine treatment. A new scoring system should include conventional symptoms and individuals' immunological parameters to provide a better guide to decrease IVIG resistance and increase effectiveness of the additional anti-inflammatory therapy. In addition to hyperinflammation and shock syndrome, patients with KDSS (20–22) or MIS-C (9, 19) usually manifest with coagulopathy, embolism, and thrombosis. It remains to be determined whether the embolism, thrombosis, and/or coagulopathy in MIS-C require certain anti-thrombotic therapy, and whether patients with MIS-C with coronary involvement require a long term aspirin treatment, which is a regimen for treating patients with KD who develop coronary abnormalities (42, 93).

## Mechanistic Immunotherapies of MIS-C Based on Infection Immunity and Autoimmunity

**Figure 1:**
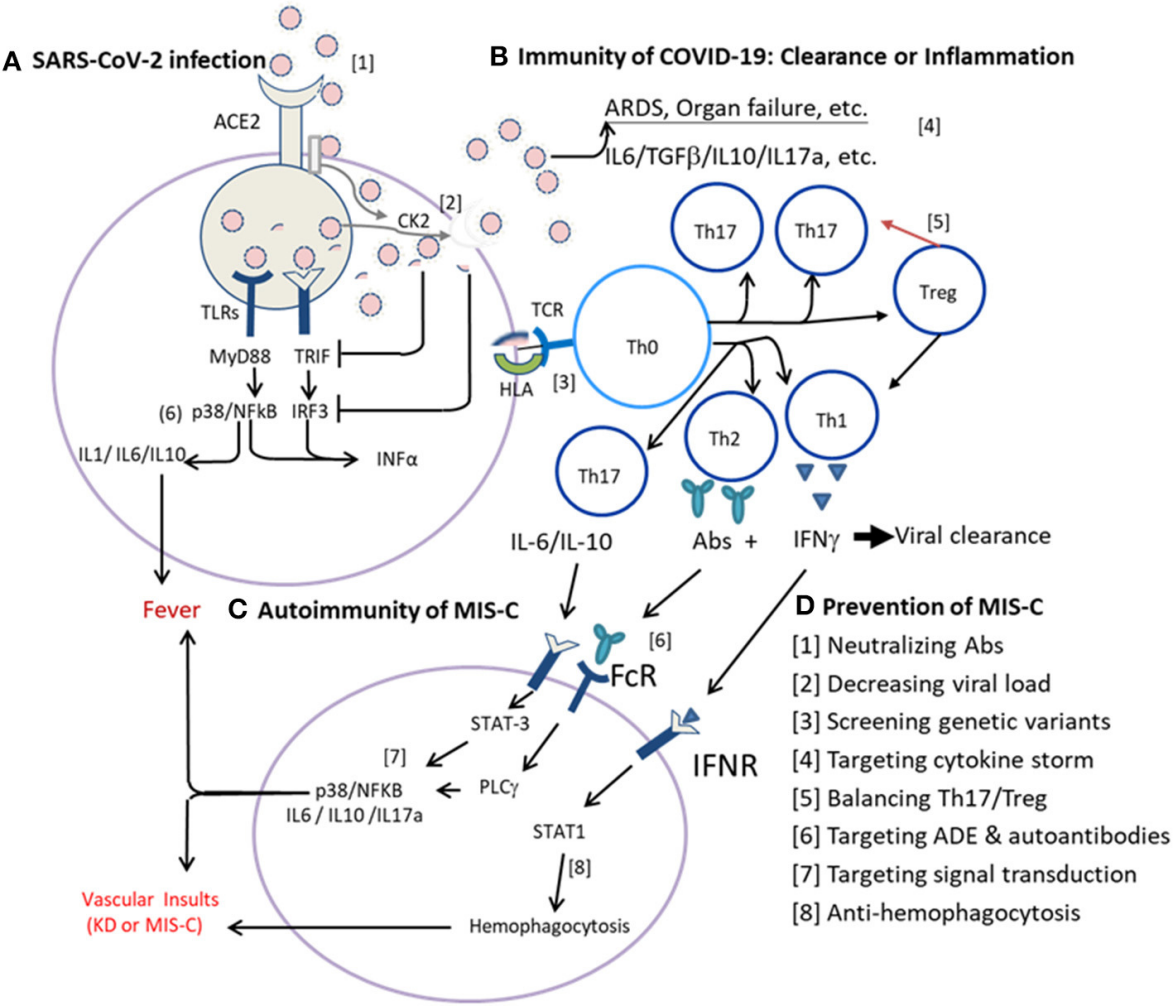
Immunotherapies of multisystem inflammatory syndrome in children (MIS-C) based on infection immunity and autoimmunity. **(A)** Innate immunity of severe acute respiratory syndrome coronavirus-2 (SARS-CoV-2) infection to cells *via* ACE2. The SARS-CoV-2 virus enters host cells *via* ACE2 where a serine protease cleaves the viral spike (S) protein and allows the virus to fuse with the plasma membrane for internalization. Host RNA sensing receptors, such as RIG-1 (DDX58), Toll-like receptor-3 (TLR3), TLR7, and/or TLR8, detect the internalized virus and induce the production of interferons (IFNs) *via* MyD88, TRIF (TICAM1), IRF3, and/or IRF7 pathways, promote production of proinflammatory cytokines *via* MAPK (e.g., p38) and NFkB pathways. Normally, the host RNA sensing receptor(s) of RIG-1 and TLR7 signaling pathways will mediate an effective induction of IFNs for virus clearance. While the virus hijacks RNA sensing receptors and pathways or activates casein kinase 2 (CK2) for filopodial protrusion of budding viral particles, the virus multiplies rapidly and the infection spreads systemically. **(B)** Adaptive immunity of coronavirus disease-19 (COVID-19). Upon antigen presentation for T-cell adaptive immunity *via* human leukocyte antigen (HLA), an optimal adaptive immunity with T-cell immunity and B-cell production of neutralizing antibodies (Abs) for virus clearance is normally elicited. While the initial virus load is high, or the TLR is either of congenital deficit or of acquired deficit as in the elderly, or the viral glycoproteins (antigens) could suppress the MyD88, TRIF, IRF3, and/or IRF7 signaling pathways, the antigen-presenting cells (APCs) are hijacked and present the viral antigen with an altered signal for the polarization of naïve T helper cells (Tho) toward Th17 response with inflammatory cytokines production, but not Treg regulation for a proper Th1 cell immunity and/or Th2 humoral (B cell) response for neutralizing antibody production. **(C)** Individual autoimmunity with MIS-C. In convalescence, most patients recovered due to efficient adaptive immunity of Th1 cell immunity and Th2 neutralizing Abs unless individual subjects with genetic susceptibility or altered endogenous milieu. For instance, the viral antigens (PAMPs) interact with individual HLA subtype(s) of APCs and induce altered autoimmunity with Th17/Treg imbalance and augmented cytokine storm of IL-6, IL-17A, and/or IL-10 expression or abnormal autoantibodies resulting in MIS-C with systemic vasculitis, thrombosis, and shock, as seen in MIS-C. In addition, certain host milieu, e.g., abnormal homeostasis of vitamins and microbiota which could compromise Treg responses and enhance Th17/Treg imbalance. Alternatively, altered FcγR or subneutralized IgG antibodies might induce antibody-dependent enhancement (ADE) of immune reaction, and autoantibodies to form immune complex or to bind endothelial cells and induce abnormal hyperinflammation. In summary, the autoimmune vasculitis of KD or MIS-C can be mediated by a genetic variant of HLA, FcγR, and/or ADE resulting in hyperinflammation with Th17/Treg imbalance. **(D)** Prevention of MIS-C can be made based on infection immunity and autoimmunity described above. A series of sequential steps ([1]–[8]) as indicated can be utilized to prevent the life-threatening MIS-C.

Until July 2020, more than 1,000 cases of MIS-C had been reported (107). However, the definition and treatment regimens are not standardized yet! While COVID-19 remains pandemic, MIS-C cases will increase further. It is also a concern that this augmentation of Th17/Treg imbalance in MIS-C after COVID-19 may be extended by a COVID-19 mass vaccination in which the vaccine antigen with adjuvant may increase the risk of MIS-C. Both KD and MIS-C are non-contagious but potentially virus or antigen- (PAMP-) induced autoimmunity in genetically susceptible individuals. Patients from different genetic backgrounds and environments including pre-existing subneutralized antibodies or abnormal autoantibodies directed against different compartments, such as endoglin, EDIL3, and or casein kinases of endothelial cells, and so on, may influence the development of MIS-C or KD (108). Both patients with MIS-C and KD have augmented IL-6, IL-17A, and IP-10 production (27, 28, 45, 108), but the levels of SCF, TWEAK, and ADA significantly decreased in patients with KD but not in patients with MIS-C (108), suggesting they have similar immune activation pathways but different regulatory (suppressive) pathways. Taken together, we postulate immunopathogenesis of the COVID-19 associated MIS-C begins with innate immunity of SARS-CoV-2 infection to cells *via* ACE2 (Figure 1A), followed by adaptive immunity of COVID-19 with antigen presentation through HLA for T-cell differentiation toward an effective immunity or altered Th17 response (Figure 1B), and leading to individual autoimmunity with MIS-C in the convalescent stage (Figure 1C).

As shown in Figure 1A, the SARS-CoV-2 virus enters the host cells *via* ACE2 where a serine protease cleaves the viral spike (S) protein and allows the virus to fuse with the plasma membrane for internalization (109). Host RNA sensing receptors, such as RIG-1 (DDX58), TLR3, TLR7, and/or TLR8, detect the internalized virus, induce IFN production *via* MyD88, TRIF (TICAM1), IRF3, and/or IRF7 pathways, and promote the production of proinflammatory cytokines *via* MAPK (e.g., p38) and NFkB pathways (110, 111). A virulent virus can hijack RNA sensing receptors and pathways or activate casein kinase 2 (CK2) for the enhancement of budding viral particles (112). While the initial virus load is low or the virus belongs to a less virulent strain, the host RNA sensing receptor(s) of RIG-1 and TLR7 signaling pathways will mediate an effective induction of IFNs and antigen presentation for an optimal adaptive immunity with T-cell immunity and B-cell production of neutralizing antibodies (Abs) for virus clearance. Alternatively, while the virus load is high, or the TLR7 is either of congenital deficit (88) or of acquired deficit as in the elderly (113), or the viral glycoproteins (antigens) could suppress the MyD88, TRIF, IRF3, and/or IRF7 signaling pathways (111, 114), the antigen-presenting cells (APCs) are hijacked and present the viral antigen with an altered signal for the polarization of naïve Th cells (Tho) toward Th17 response with inflammatory cytokines production, but not Treg regulation for a proper Th1 cell immunity and/or Th2 humoral (B cell) response for neutralizing antibody production. The virus will multiply effectively, and a large number of antigens (PAMPs) will be released to augment Th17/Treg imbalance and promote cytokine storm, leading to ARDS with neutrophilia and lymphopenia, epithelial cell damage, vascular leakage, and/or coagulopathy in COVID-19 (Figure 1B).

In convalescence (Figure 1C), most patients recovered due to efficient adaptive immunity of Th1 cell immunity and Th2 neutralizing antibodies. While the viral antigens (PAMPs) interact with certain HLA subtype(s) of APCs and induce altered autoimmunity with Th17/Treg imbalance, in which an augmented cytokine storm of IL-6, IL-17A, and/or IL-10 expression and abnormal autoantibodies could result in MIS-C with systemic vasculitis, thrombosis, and shock as seen in MIS-C. The abnormal virus-host response may not only depend on genetic variants, e.g., HLA subtypes (87) and TLR7 variants (88), but also host milieu, e.g., homeostasis of vitamins and microbiota which could maintain better Treg responses for anti-inflammatory reactions (115–117). Alternatively, altered FcγR or subneutralized IgG antibodies might induce ADE of immune reaction. ADE resulting from the interaction of the FcγRIIA with a variant polymorphism had been found in SARS-CoV-1 infections (118), and a similar mechanism has been demonstrated *in vitro* in COVID-19 (119). It is possible that the autoimmune vasculitis of KD or MIS-C is mediated by a genetic variant of HLA, FcγR, and/or ADE resulting in hyperinflammation with Th17/Treg imbalance. However, the Th17/Treg imbalance may be different between MIS-C and KD because the Th17 mediators were elevated in both the diseases but the immunosuppressive mediators: SCF, TWEAK, and ADA were lower in KD than in MIS-C (108). In patients with KD or MIS-C with failure of IVIG and corticosteroids treatment, additional immunotherapies might be applicable by targeting different Th17/Treg imbalances.

### Therapeutic Perspectives of MIS-C

Based on the postulated immunopathogenesis of COVID-19 associated MIS-C described above, we could make a series of sequential steps ([1]–[8]) to prevent the life-threatening MIS-C as indicated in Figure 1D and as described below:

1. *Blocking virus entry by neutralizing Abs*. In a meta-analysis of 12 controlled trials with more than 4,000 participants, transfusions of convalescent plasma with neutralizing Abs interrupted the virus-ACE2 interaction. The treatment in hospitalized COVID-19 patients reduced the mortality rate by 57% (10 vs. 22%; OR: 0.43, *p* < 0.001) (120). Similarly, convalescent plasma or neutralizing monoclonal antibodies (MoAbs) have also rescued patients with Ebola (121), SARS (122), and Middle East respiratory syndrome (MERS) (123). Thus, early administration of hyperimmune or recombinant MoAbs with neutralizing Abs directed against SARS-CoV-2 should be able to decrease virus load and raise better immune response toward balanced Th17/Treg reaction resulting in less severity and less autoimmunity.
2. *Decreasing viral load*. There are many *in vitro* studies showing that several potential anti-COVID-19 agents could block the entry, replication, and/or shedding of SARS-CoV-2 (112, 124). The decrease of viral replication and shedding could be made by the inhibition of virus-cell fusion, virus and host proteases, lysosome acidification, RNA synthetase, and virus budding (124, 125). A proper regimen (e.g., remdesivir, avigan, or silmitasertib) to decrease the virus transmission between the infected and non-infected cells may enhance immune response and mitigate the possible autoimmunity. A combination of neutralizing MoAbs and anti-virus agent may induce a synergistic effect.
3. *Screening genetic variants*. Similar to KD which has been linked to certain alleles of HLA subtypes in regard to disease susceptibility and severity, the severity of COVID-19 has been proposed to be associated with HLA-B^*^46:01 in a computational simulation by using SARS-CoV-2 whole-genome peptides for simulating their binding to 145 MHC class I HLA-A, -B, and -C genotypes (87). Moreover, a recent report showed that a mutant (D839Y/N/E) from a European strain of SARS-CoV-2 could serve as a superantigen to induce T-cell receptor activation, resulting in hyperinflammatory response, which may be implicated in the development of MIS-C as well as cytokine storm in adult patients with COVID-19 (126). Deletion or mutation of TLR7 has also been attributed to more severity of COVID-19 in young adults (88). Further studies to identify the risk genetic variants for severity and/or autoimmunity of COVID-19 would help develop a screening genetic test for protecting susceptible children from contacts of COVID-19 and incubate better Th17/Treg balance by nurturing internal milieu with proper homeostasis of vitamin D, vitamin A, and microbiota (104–106).
4. *Targeting cytokine storm*. In an early trial with anti-IL6R for patients with COVID-19 hospitalized with cardiopulmonary exacerbation showed potential benefits in decreasing CRP levels, fever, and severity (127). However, later randomized trials demonstrated no significant effects on the severity or fatality of COVID-19 (128). Taken together, aiming at a single target of one cytokine action may be ineffective but a combined regimen or sequential targeting may be required for eliminating the cytokine storm mediated by a couple of hyperinflammatory cytokines in COVID-19 or MIS-C. Th17 mediators, Il-6 and IL-17A and Th1 down-stream mediators, TNF-α and IP-10, more prominently increased in KD than in MIS-C (27, 28, 45, 108), suggesting that targeting IL-17A by Secukinumab or anti-TNFα could be considered in patients with KD with IVIG resistance or with KDSS. Children with MIS-C, who did not have IL-17A or TNFα overexpression (108), may be treated with a combination of IVIG, corticosteroids, and recombinant IL-1-receptor antagonist, Anakinra.
5. *Balancing Th17/Treg immune response*. Abnormal immune regulation has been shown in patients with KD or MIS-C (46–50). Both genetic and epigenetic alterations in Treg pathways have been demonstrated in patients with KD (53– 56). The induction and/or stabilization of Treg cell development is affected by endogenous milieu, such as vitamins and metabolites from microbiota (115– 117, 129–131). Vitamin D levels have been shown lower in many patients with COVID-19 and associated with increased inflammatory cytokines and an increased risk of pneumonia (129). The lower vitamin D concentration is not only linked to higher severity of COVID-19 (130, 131), but also associated with an increase in thrombotic episodes (132, 133), which are frequently observed in COVID-19 associated MIS-C (4–9). Vitamin D deficiency has been also shown to be associated with KD with IVIG resistance (134). Moreover, microbiota have recently been shown to coordinate adipocyte-derived mesenchymal stem cells to combat autoimmunity of Type 1 diabetes in mice (135), and mesenchymal stem cells (MSC) or their exosomes have been proposed to eliminate hyperinflammation of COVID-19 (136, 137). We have also recently shown that exosomes derived from MSCs (MSC-EVs) could rescue inflammatory neuropathic pain (138). Evidence accumulated has demonstrated that the effects of MSCs and exosomes derived from MSCs are useful in treating inflammatory diseases and fibrosis (139, 140). This regimen may be suitable for not only cytokine storm but also post-infectious pulmonary fibrosis. In addition, epigenetic modulations of FOXP3 expression by DNA methylation and/or miRNA expression (54–60), may also be applicable to correct the Th17/Treg imbalance.
6. *Targeting ADE and autoantibodies*. ADE of immunopathology has been concerned to potentially happen in dengue virus, Zika virus, Ebola virus, respiratory syncytial virus (RSV), and coronaviruses (119). The potential ADE in MIS-C could be treated by using IVIG with and without corticosteroids as shown in patients with MIS-C (4–9), or might be rescued by the elimination of the glycosylation site at N297 of the IgG Fc portion or by a mutation in the Fc region resulting in an effective antibody neutralization but not ADE (119). Several autoantibodies, such as autoantibodies to MAP2K2, CSNK1A1, CSNK2A1, and CSNK1E1 were notably found in patients with MIS-C, and autoantibodies directed against EDIL3 were exclusively found in patients with KD (108), suggesting these autoantibodies might be used as biomarkers for differential diagnosis and their anti-idiotypic antibodies might be used for prevention of autoimmune vasculitis.
7. *Targeting signal transduction pathways*. COVID-19 has been shown to induce hyperactivation of TLR-mediated MAPK pathway and CK2-mediated filopodial protrusion of viral shedding (112). Inhibition of p38 activation has been shown to decrease viral replication and cytokine induction in *in vitro* cell model (112). This is further supported by a recent report showing autoantibodies to MAP2K2, and three members of the casein kinase family (CSNK1A1, CSNK2A1, and CSNK1E1) are notable in children with MIS-C (108). Inhibitors of the phosphokinases which are activated in an *in vitro* model of SARS-CoV-2 infection, including CK2, CDK, AXL, and PIKFYVE kinases, may possess antiviral efficacy. A combination of different inhibitors of the kinases may have a synergistic effect on anti-viral and anti-inflammatory responses. A recent study showed a combination of a viral protease inhibitor, GC376, and the RNA-dependent RNA synthetase inhibitor, remdesivir, offered sterilizing additive effects (125). In addition, a proteasome inhibitor MG132 which could inhibit IL-6/TGF-β-mediated downregulation of FOXP3 protein may potentially raise the Treg activity (60).
8. *Anti-hemophagocytosis*. Hemophagocytosis syndrome also called MAS usually occurs in patients with autoimmune disorders (141, 142). Interestingly, patients with KDSS (20–22) or MIS-C (9, 19) can have IVIG resistance associated with the hemophagocytosis with anemia, elevated Th1 mediator, such as IFNγ, associated with hyperferritinemia and hypertriglyceridemia. In this situation, a combination of IVIG with cyclosporin-A, anti-TNF-α, and/or MSC administration may be required (9, 30, 98–101).

In summary, the autoimmune vasculitis of KD, KDSS, or MIS-C is mediated by a genetic variant of HLA, FcγR, and/or ADE resulting in hyperinflammation with Th17/Treg imbalance with augmented Th17/Th1 mediators: IL-6, IL-10, IP-10, IFNγ, and IL-17A, and a lower expression of Treg-signaling molecules, FoxP3 and TGF-β, and other suppressive immune mediators. Th17/Treg imbalances among them share similar activation pathways but different regulatory (suppressive) pathway. Based on the similar and different immunopathogenesis, we can make early protection, prevention, and precision treatment of the diseases beyond IVIG and corticosteroids therapies. Evolution of immunotherapies for the diseases has shown that IVIG alone or combined with corticosteroids is the standard treatment for KD, KDSS, and MIS-C. However, some patients are resistant to these therapies, and these susceptible individuals must be detected and given the treatment which can render an early block of viral entry, viral replication and/or shedding, and combat Th17/Treg imbalance by anti-cytokine or pro-Treg for reversing the hyperinflammation and IVIG resistance. Clarifying phenotypes, genetic susceptibility, and hyperinflammatory mechanisms of KD, KDSS, and MIS-C with and without IVIG resistance may help develop a so-called “Know thyself, enemy (pathogen) and ever-victorious” strategy for prevention and immunotherapy for KD and/or MISC.

## Data Availability Statement

The original contributions presented in the study are cited properly in the references, further inquiries can be directed to the corresponding author/s.

## Author Contributions

M-RC collected the references and summarized the KD studies in Mackay Children's Hospital for drafting the manuscript. H-CK collected the references and summarized the KD studies in Kaohsiung Chang Gung Memorial Hospital for drafting the manuscript. Y-JL drafted the scheme and revised the manuscript. HC provided the information and references regarding KDSS and compared the phenotypes among KD, KDSS, and MIS-C. SL provided the references and suggestions for the section regarding epigenetic controls of Th17/Treg balance on the autoimmunity of KD and MIS-C. H-CL drafted the section of Treg balance influenced by milieu conditions, such as homeostasis of vitamin D and microbiota. KY designed the article scheme and organized the information for the completion of the article approved by all authors before submission. All authors contributed to the article and approved the submitted version.

## Conflict of Interest

The authors declare that the research was conducted in the absence of any commercial or financial relationships that could be construed as a potential conflict of interest.
