# Corrigendum: Phenotype, Susceptibility, Autoimmunity, and Immunotherapy Between Kawasaki Disease and Coronavirus Disease-19 Associated Multisystem Inflammatory Syndrome in Children

**DOI:** 10.3389/fimmu.2021.722582

**Published:** 2021-08-05

**Authors:** Ming-Ren Chen, Ho-Chang Kuo, Yann-Jinn Lee, Hsin Chi, Sung Chou Li, Hung-Chang Lee, Kuender D. Yang

**Affiliations:** ^1^MacKay Children’s Hospital, Taipei, Taiwan; ^2^MacKay Junior College of Medicine, Nursing, and Management, New Taipei City, Taiwan; ^3^Kawasaki Disease Center and Department of Pediatrics, Kaohsiung Chang Gung Memorial Hospital, Kaohsiung, Taiwan; ^4^Genomic and Proteomic Center, Kaohsiung Chang Gung Memorial Hospital, Kaohsiung, Taiwan; ^5^Department of Microbiology & Immunology, National Defense Medical Center, Taipei, Taiwan; ^6^Institute of Clinical Medicine, National Yang Ming University, Taipei, Taiwan

**Keywords:** Kawasaki disease, multisystem inflammatory syndrome in children, susceptibility, autoimmunity, immunotherapy, coronavirus disease-19

An author name was incorrectly spelled as **Men-Ren Chen**. The correct spelling is **Ming-Ren Chen**.

The authors apologize for this error and state that this does not change the scientific conclusions of the article in any way. The original article has been updated.

## Publisher’s Note

All claims expressed in this article are solely those of the authors and do not necessarily represent those of their affiliated organizations, or those of the publisher, the editors and the reviewers. Any product that may be evaluated in this article, or claim that may be made by its manufacturer, is not guaranteed or endorsed by the publisher.

